# Commentary on: The Best Under Stress: An Analysis of Breast Tissue Expander Response to External Forces

**DOI:** 10.1093/asjof/ojad020

**Published:** 2023-02-23

**Authors:** Maurice Y Nahabedian

This experimental study was designed to test the deformational properties of 3 commercially available breast tissue expanders distributed by Mentor (Irvine, CA), Allergan (Irvine, CA), and Sientra (Santa Barbara, CA).^1^ The purpose was to determine if various compressive loads, when applied to a fully filled saline tissue expander, would result in deformation based on base width, projection, and height. The implication being that this could affect clinical outcomes. The details of the study are well described in the manuscript. The authors found that the Mentor device had the least amount of deformation for all parameters tested at the various forces applied (Video).

This is an interesting concept to study, but my question is whether or not it makes any “clinical” difference based on current techniques of tissue expander breast reconstruction? Following mastectomy, the majority of plastic surgeons now place tissue expanders in the prepectoral space where compressive forces are minimized. Prior to the prepectoral era, tissue expanders were placed in the subpectoral space where compressive forces were markedly increased due to the repeated contraction of the pectoralis major muscle. These forces predisposed prosthetic devices toward inferior or lateral displacement. Studies have demonstrated that the incidence of implant displacement in prepectoral reconstruction is ∼5.9%^2^ and in subpectoral breast reconstruction is ∼9.8%.^3^ As a means of overcoming these compressive forces, overfilling of tissue expanders was often necessary with subpectoral placement. These compressive forces were exacerbated following radiation therapy due to the progressive fibrosis of the pectoralis major muscle and the overlying adipocutaneous layer. To minimize the risk of device malposition, meticulous suturing techniques were necessary to strictly define the breast footprint. With prepectoral reconstruction, the external forces applied to the tissue expander are minimized since the tissue expander sits on top of the pectoralis major muscle. Although the overlying skin can apply some compression to the tissue expander, the effect is far less than that of the pectoralis major muscle. In addition, the adaptation of the soft tissues to stretch is facilitated based on the process of creep and stress relaxation. With the advent of tabbed tissue expanders, the tissue expander is sutured to the chest wall so displacement rarely occurs.^4^ As a result of the compliant overlying soft tissues, overfilling of the tissue expander is rarely necessary.

This study attempts to emphasize the concept of the made-to-match phenomenon between the tissue expander and implant and suggests that it is optimized with the Mentor device due to minimal compressive deformation. It should be noted that with prepectoral placement of tissue expanders, the tissue expander does not shape or contour the skin envelop; it primarily serves as a space holder, maintains adipocutaneous stretch, and serves to define the breast footprint. The final shape of the reconstructed breast is ultimately determined by the quality of the mastectomy skin, the dimensions of the breast footprint, and the shape and cohesivity of the final implant. The ability of a tissue expander to withstand compressive forces is no longer a determinant of final outcome.

In summary, it is the readers’ opinion that all tissue expanders are capable of delivering optimal clinical outcomes in the setting of prepectoral reconstruction. The forces applied to a tissue expander placed in the prepectoral position are far less than what was applied to the expander for the purposes of this study. Thus, the choice of tissue expander should be based on ergonomics and surgeon preference.

## Supplementary Material

ojad020_Supplementary_Data
